# Benefits of Exercise in Multidisciplinary Treatment of Binge Eating Disorder in Adolescents with Obesity

**DOI:** 10.3390/ijerph19148300

**Published:** 2022-07-07

**Authors:** Hellas Cena, Matteo Vandoni, Vittoria Carlotta Magenes, Ilaria Di Napoli, Luca Marin, Paola Baldassarre, Alessia Luzzi, Francesca De Pasquale, Gianvincenzo Zuccotti, Valeria Calcaterra

**Affiliations:** 1 Laboratory of Dietetics and Clinical Nutrition, Department of Public Health, Experimental and Forensic Medicine, University of Pavia, 27100 Pavia, Italy; hellas.cena@unipv.it (H.C.); ilaria.dinapoli@unipv.it (I.D.N.); francesca.depasquale01@universitadipavia.it (F.D.P.); 2 Clinical Nutrition and Dietetics Service, Unit of Internal Medicine and Endocrinology, ICS Maugeri IRCCS, 27100 Pavia, Italy; alessia.luzzi@unimi.it; 3 Laboratory of Adapted Motor Activity (LAMA), Department of Public Health, Experimental Medicine and Forensic Science, University of Pavia, 27100 Pavia, Italy; matteo.vandoni@unipv.it; 4 Pediatric Department, “Vittore Buzzi” Children’s Hospital, 20154 Milan, Italy; vittoria.magenes@unimi.it (V.C.M.); paola.baldassarre@unimi.it (P.B.); gianvincenzo.zuccotti@unimi.it (G.Z.); 5 Research Department—LJA 2021, Asomi College of Sciences, 2080 Marsa, Malta; luca.marin@unipv.it; 6 Department of Rehabilitation, Città di Pavia Hospital, 27100 Pavia, Italy; 7 Post Graduate Course in Food Science and Human Nutrition, Università Statale di Milano, 20122 Milan, Italy; 8 Department of Biomedical and Clinical Science, University of Milan, 20157 Milan, Italy; 9 Department of Internal Medicine, University of Pavia, 27100 Pavia, Italy

**Keywords:** binge eating, obesity, exercise, physical activity, children, adolescents

## Abstract

Obesity in childhood and adolescence represents a serious health problem worldwide. Similarly, eating disorders (EDs) are complex diseases that affect adolescents with an increasing prevalence and are an alarming health concern to both physical and mental health. Traditionally, obesity and EDs, particularly binge eating disorder (BED), have been considered separate conditions, but there is emerging evidence such as etiology, comorbidities, risk factors, psychosocial impairment, and prevention approaches, highlighting important overlaps among these conditions. In youth, the two conditions share risk factors and consequences at both the physical and psychological levels, requiring special care. Exercise, useful as strategy to prevent and treat overweight conditions, may have beneficial effects on BED symptoms, suggesting that it may be considered as one of the key factors in the treatment of individuals affected by obesity with BED. The purpose of this narrative review is to examine the bidirectional impact of obesity and BED in adolescents, in terms of risk factors, etiology and comorbid conditions. Specifically, we focused on the benefits of physical activity (PA) in the multidisciplinary treatment of subjects affected by obesity with BED. Even though additional research is needed to reach conclusions about the role of exercise in the treatment of obesity and comorbid BED, especially in adolescents, promising results have already suggested that closely monitored exercise is safe and, paired with cognitive behavioral therapy, may provide multiple benefits on both the physical and psychological levels. Tailored and integrated treatments for weight management and eating disorders are important to promptly and effectively treat obese subjects that have BED.

## 1. Introduction

Adolescence is a period of growth and development where children undergo significant physical, intellectual, and emotional changes. It represents a critical transition to adulthood, and it is characterized by significant changes in body size and composition.

During adolescence, a healthy lifestyle is crucial to adequately grow and avoid excessive fat mass accumulation and its known negative health effects.

In this extremely delicate period of life, both obesity and eating disorders represent serious health problems worldwide [1,2].

Obesity, for which the rate in the population has dramatically increased in the last few decades, is a pathological health condition characterized by excessive adipose tissue storage because of an imbalance between caloric intake and energy consumption. Obesity is responsible for serious obesity-related comorbidities and profoundly affects physical health, social, and emotional well-being, and self-esteem in children and adolescents [1,2,3,4,5].

Similarly to obesity, EDs are complex diseases that affect adolescents with an increasing prevalence [1] and are an alarming health concern to both physical and mental health [2,3].

Traditionally, obesity and EDs, particularly binge eating disorder (BED), have been considered separate conditions, but there is emerging evidence—such as etiology, comorbidities, risk factors, psychosocial impairment, and prevention approaches—highlighting important overlaps among these conditions [2,3]. Indeed, these two pathological conditions have a bidirectional impact [2]. On one hand, BED is considered a risk factor for obesity development, on the other, obesity may increase the risk for BED [3]. Thus, the prompt recognition and a correct approach of BED may help both conditions, preventing obesity development and/or promoting weight loss in overweight or patients affected by obesity [2].

The combination of physical and emotional comorbidities and the multifactorial etiology present in obesity and BED suggests the importance of a multidisciplinary approach to management. A multidisciplinary integrated approach should include nutritional education, physical activity (PA) and behavioral interventions, which have been acknowledged as the treatment of choice in all EDs, including BED [6]. Moreover, this multidisciplinary approach, to be effective, has to address both physical and psychological aspects of the two comorbid pathologies addressing being overweight or obese and enabling adolescents to positively react to emotional stress and external stimuli, decreasing binge eating episodes [6]. In this context, PA, if accurately tailored on patient needs, may have a crucial role.

Over the last few years, a sedentary lifestyle has become increasingly common within the general population [7], and, during the current pandemic, a concurrent increase of overweight, unhealthy eating dietary patterns, isolation, mood deflections, and eating disorders prevalence was also observed, especially in adolescents [8].

A study by Guthold et al. (2020), found that more than 80% of school-going adolescents globally did not meet current recommendations of at least one hour of physical activity per day—including 85% of girls and 78% of boys. Indeed, PA and sports engagement have an important positive impact on health, well-being, and psychosocial fitness in children and adolescents. As shown by Carr, PA is a significant factor differentiating adolescents with obesity and BED from those with obesity without BED [9,10]. Therefore, exercise may be considered as one of the key factors in the treatment of individuals affected by obesity with BED, simultaneously improving both physical and psychological health [9].

Tailored and integrated treatments for weight management and eating disorders in individuals with obesity and BED are important to treat subjects promptly and effectively for obesity and BED. The purpose of this narrative review is to examine the bidirectional impact of obesity and BED in adolescents, in terms of risk factors, etiology and comorbid conditions. Specifically, we focused on the benefits—physical and psychological—of PA in the multidisciplinary treatment of subjects affected by obesity with BED. The integrated treatment, including dedicated exercise programs, may be useful to provide tailored therapeutic strategies.

## 2. Methods

A narrative review of the literature was conducted [9], presenting a non-systematic summation and analysis of available literature on the topic of PA and exercise benefits in the multidisciplinary treatment of binge eating disorder in adolescents with obesity. To refine the scope of our narrative review, we established a set of inclusion criteria: articles published in the last twenty years, up to May 2022, in English language; original scientific papers, clinical trials, meta-analyses and reviews published on a specific topic; articles on pediatric patients and adults. Case reports or series and letters were excluded. The authors assessed the abstracts of the available literature (*n* = 134) and reviewed the full texts of potentially relevant articles (*n* = 76) that were analyzed to provide a critical discussion. In addition, the reference list of all articles was checked to identify relevant studies. The research terms adopted, alone and/or combined, are eating disorders, obesity, adolescents, binge eating disorder, exercise, aerobic exercise, resistance exercise, PA, therapy, multidisciplinary treatment, and multidisciplinary approach. The databases PubMed, Scopus, EMBASE and Web of Science were used (from February to May 2022) for research purposes. The contributions were independently collected by V.C.M., I.D.N., P.B., A.L., F.D.P. and critically analyzed with H.C., V.C., M.V. and L.M. The resulting draft was discussed by H.C., V.C., M.V. and critically revised by V.C. and G.Z. The final version was then recirculated and approved by all.

## 3. Adolescents with Obesity

**Figure 1:**
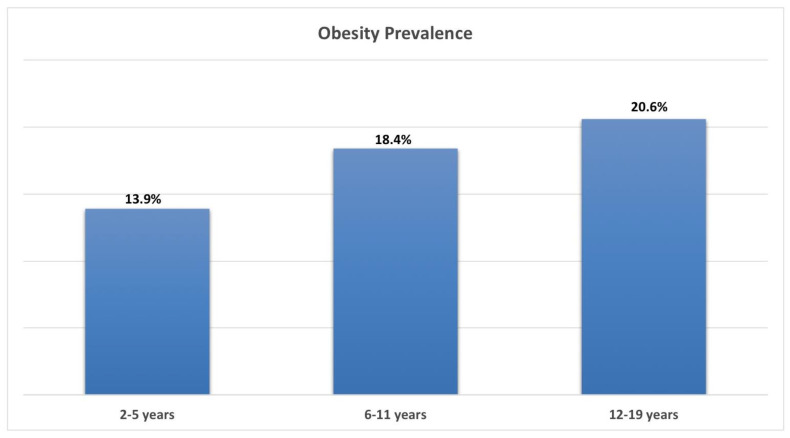
Obesity prevalence in children and adolescents.

Obesity in childhood and adolescence represents an emerging worldwide health problem characterized by excessive adipose tissue storage because of an imbalance between caloric intake and energy consumption. Over the last few decades, the obesity pandemic has dramatically increased, having doubled since 1980. It is worth mentioning that even developed countries have been affected by this increase [10,11]. The prevalence of obesity is higher in adolescents between 12 and 19 years (about 20.6%); as for younger children aged 6–11 years and 2–5 years, obesity is at about 18.4 and 13.9%, respectively [12,13,14], Figure 1.

According to WHO estimates, in 2016, 340 million children and adolescents between 5 and 19 years were diagnosed as overweight or obese. In 2019, 38.2 million children under 5 years received the same diagnosis. The prevalence of this emergent disease among children and adolescents aged 5–19 has risen dramatically from 4% in 1975 to over 18% in 2016, where 18% of girls and 19% of boys were overweight [15].

Obesity is a condition characterized by an interaction of multiple and complex factors and predispositions, most of which are still poorly understood. It promotes an inflammatory state that is strongly associated with many comorbidities involving almost all body systems and organs, increasing the risk of chronic disease development in adolescence and, consequently, in adulthood [16]. Studies have evidenced that about 80% of adolescents with obesity remain affected by this disease into their adult lives, leading to increased morbidity and mortality [17].

Most cases of obesity are polygenic as they affect the entire phenotype on which several environmental and behavioral factors act. In contrast, monogenic obesity concerns only about 3 to 5% of children affected by obesity, and in these cases weight gain develops mostly in the first years of life. The most common gene defect is a mutation that involves the melanocortin 4 receptor gene (MC4R), which is linked to a more severe and early form of obesity [17,18,19]. Epigenetic factors may also modify the interaction of the environment, microbiome, and nutrition in promoting weight gain.

In industrialized societies, adolescents are more exposed to obesogenic environmental factors and to emotional and psychological stress. It is well known that cultural and socioeconomic environments play a bigger role in the development of adolescent obesity than genetic factors [20].

The steady rise in obesity incidence is partially caused by changes in lifestyle habits and especially the great availability, affordability, and frequent consumption of fast food and sugar-sweetened beverages, which is also associated with an increase in BMI [21]. Sedentary behaviors and lack of PA are other important risk factors. In fact, several studies in the literature have demonstrated the close link between obesity and screen time: watching television, playing video games, or using computers [22].

Moreover, obesity, as per the literature, starts in utero. Intrauterine exposure to maternal obesity, physical inactivity, diet, smoking, excessive gestational weight gain, and gestational diabetes are all factors that may lead to the development of obesity [22,23,24].

Adolescents suffering from obesity may experience its harmful health effects, including metabolic complications such as insulin resistance, hyperglycemia, diabetes, and cardiovascular outcomes such as dyslipidemia, hypertension, and endothelial dysfunction [25]. Therefore, persistent hyperinsulinemia determines a state of prediabetes (impaired fasting glucose and/or impaired glucose tolerance), which may become type 2 diabetes mellitus (DMT2). Additionally, a typical dermatological alteration called acanthosis nigricans, which consists of a hyperpigmentation and thickening of the skin folds, indicates insulin resistance [23]. Cardiovascular diseases are commonly classified as adulthood diseases, but studies have suggested that the atherosclerosis process starts in childhood or adolescence and its progression and evolution depend on the presence of cardiovascular risk factors in youth. Studies have established that about 70% of children and adolescents aged 5–17 years who suffer from obesity have at least one cardiovascular risk factor, and that hypertension and dyslipidemia are associated with the onset and severity of early atherosclerotic lesions in adolescents and young adults [12,25,26].

Childhood and adolescent obesity are also associated with several musculoskeletal problems such as impairment in mobility, lower extremity joint pain or misalignment and also orthopedic complications like genu valgum, tibia vara (Blount disease), and fractures [27].

As reported in literature, the distribution of fat mass in different body segments influences body geometry and, therefore, the postural control and stability in overweight or obese children, increasing the risk of falls compared to individuals with normal weight. Consequently, the individual develops adaptive mechanisms to maintain balance. In children demonstrated as having high BMI, fat percentage and waist circumference are connected with poorer motor skills. An adequate dietary behavior together with PA based on reducing trunk and leg mass, maintaining lean mass, may improve postural control and decrease impact forces during walking [28,29].

Aside from the physiological consequences, obesity in children and adolescents can also negatively impact their cognitive development, affecting personality and self-esteem. Among its psychological manifestations, many studies have shown that, compared to children without obesity, children with obesity are at a higher risk of anxiety, low self-confidence, and body dissatisfaction, which affect their daily quality of life [15,26,30]. These conditions may contribute to the onset of eating disorders and other unhealthy weight-control behaviors, sometimes leading to the extreme action of suicide. As for BED, it represents one of the most common nutritional and eating disorders in pediatric obesity. It occurs in approximately 25% of female adolescents with obesity and could be a causative factor in the development of obesity, especially in the presence of family history or other risk factors [31]. Children and adolescents affected by obesity are also bullied and subject to social shaming, which implies psychological distress and more difficulties in adulthood, increasing the risk of depression. In the age group between 3 and 17 years old, it is estimated that 3.7% (about 1.9 million) developed depression, as this psychiatric disorder derives from the isolation and stigma of obesity. Anxiety disorders are present in 7.1% (about 4.4 million) of children and adolescents aged 3–17 years old and represent the most frequent mental health disorders in children. Eating disorders particularly concern girls [31]. In short, adolescence is a stressful period, and several sources consider the control of psychosocial stress to be a fundamental aspect of the prevention and management of obesity.

**Figure 2:**
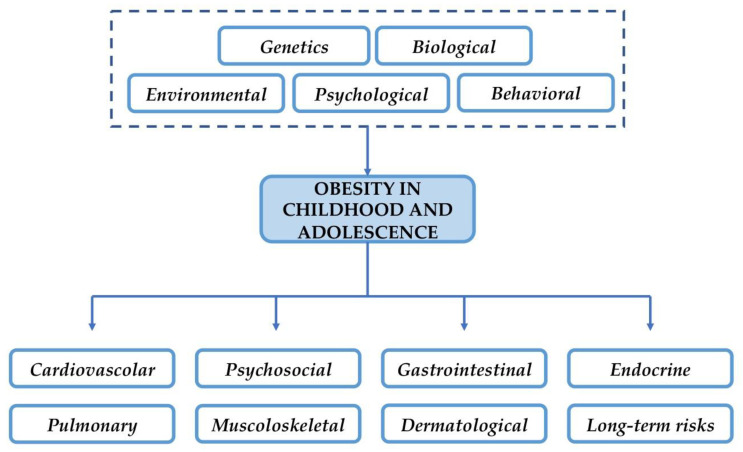
Risk factors and comorbidities in pediatric obesity.

In Figure 2, risk factors and comorbidities in pediatric obesity are reported.

The prevention of obesity in children is necessary to avoid chronic diseases and harmful repercussions for adolescent and adult life. Therefore, it is necessary to implement a multidisciplinary and multistep method to modify the current lifestyle.

Intervention strategies are based on the modification of home activities, school life, environmental and cultural practices with the help of national policies aimed at reducing emotional stress and providing adolescents with the awareness and the competence to make healthy food choices [19,27,32].

## 4. Binge Eating Disorder in Adolescents with Obesity

Among EDs, BED is the most prevalent disease in children and adolescents with obesity [33]. Binge eating disorder is a relatively new disorder and was first included as an independent diagnosis in the fifth edition of the *Diagnostic and Statistical Manual of Mental Disorders* (DSM-5) [34,35]. The main symptom of BED is the frequent occurrence of binge eating episodes [30], where a binge eating episode is defined as the consumption of an objectively large amount of food without compensatory behaviors, eaten in a discrete period of time [34,35]. Its criteria require binge eating episodes at least once a week for 3 months [35]. The episode is associated with a subjective experience of feeling the loss of control (LOC) and marked distress [35]. The term LOC eating is used to describe episodes with LOC, where the amount eaten is either not defined or not objectively large [34]. This term is extremely useful in children and adolescents, as it is challenging to define “a large amount of food” for them when considering possibilities such as increases in calories related to growth spurts [36]. The LOC eating episodes can be further subdivided into subjective binge episodes (SBEs), in which the individual experiences a loss of control and does not consume an objectively large amount of food, and objective binge episodes (OBEs), in which the patient experiences both a loss of control and consumes an objectively large amount of food [36].

There has been considerable uncertainty regarding the prevalence rate of BED or LOC eating among children and adolescents that are obese or overweight, mainly because of inconsistent findings across the studies [34,37]. Indeed, in their meta-analysis, He et al. indicated that BED or LOC eating was prevalent (i.e., slightly over a quarter of the population) among children and adolescents that were overweight or obese [37]. These results were confirmed by He et al., who estimated the prevalence of binge eating and LOC eating to be 26.3% in children and adolescents affected by being overweight or obese, and recently verified by Moustafa et al., who estimated rates of LOC eating episodes at about 31.2% in overweight youth [36]. Both BED and LOC eating are more prevalent in children and adolescents that are overweight compared to peers with normal weight [34]. Interestingly, within the adult population, the lifetime prevalence of BED varied according to body mass index (BMI): 1.2% in subjects that were overweight, and 2.6 and 4.5% in subjects that were obese with a BMI of 30–35 kg/m^2^ or BMI of ≥35 kg/m^2^, respectively [38,39]. These results suggest a direct and important correlation between BED and BMI, with BED lifetime prevalence becoming higher while BMI increases [38].

Obesity and BED are generally considered independent disorders; however, these two pathological conditions can have a bidirectional impact [31]. Indeed, a strong link exists between obesity and binge/LOC eating. On one hand, BED is regarded as a risk factor for obesity in childhood and adolescence [37,40,41]; on the other, being overweight and obesity may also increase the risk for BED, as binge eating is more likely to be associated with overweight populations [37,42]. In addition, previous studies have also suggested that LOC eating could be a salient predictor for weight gain during middle childhood and adolescence [43,44,45]. Thus, the prompt recognition of BED may help both conditions, preventing obesity and promoting weight loss in cases of sustained obesity [31,46]. Indeed, it was found that 62.8% of patients with BED had a family history of obesity and almost 29.0% were children or adolescents with obesity [38,47]. Furthermore, a family history of BED is considered a risk factor for the development of obesity later in life [38,48].

In addition, specific socio-environmental and individual conditions are considered shared risk factors for BED and obesity [38]. Among environmental factors, the more frequently reported are family and peer teasing, social pressure, and bullying [49]. These factors contribute to body dissatisfaction, further enhanced by television or social media images focused on the ideals of slimness and beauty [50]. Regarding individual risk factors, these can be subdivided into biological/biochemical and psychological/behavioral factors. Among biological ones, genetics plays a role. The most known susceptibility gene for obesity is the fat mass and obesity-associated (FTO) gene [51]. Variants of this gene have also been associated with BED, suggesting a genetic role in the pathogenesis of this disorder [52]. Among biochemical risk factors, hormones involved in the regulation of satiety and hunger have a role in both obesity and EDs [38]. Specifically, ghrelin, an appetizing hormone, and leptin, a promoter of the feeling of satiety, are important factors in weight control [53]. Often associated with obesity, these hormones have also been implicated in the pathophysiology of EDs [53,54,55]. Concerning psychological risk factors, low self-esteem, negative self-evaluation, depression, and body dissatisfaction are considered to be contributors to the development of both EDs and obesity [31,44,56,57,58]. In terms of behavioral factors, the most described is dieting [31,38]. Indeed, it is well known that dieting increases the risk of overeating to counteract the caloric deprivation and, over time, results in weight gain [38,57,59]. The “dietary restraint theory” is based on the premise that caloric restriction increases the risk of BE when individuals tend to have cognitive control over eating behaviors [59]. Patients subjected to a strict dietary regulation respond to any transgression of the diet with an all-or-nothing response that results in a binge episode [37,56,57]. Moreover, dieting to compensate for the effect of binge eating triggers additional binges. This ultimately leads to repeated cycles of dieting and binge eating [59,60,61]. This phenomenon is further enhanced by some of the above-described psychological risk factors (such as body dissatisfaction, depression, perceived pressure to be thin or thin-ideal internalization) that promote dieting and increase the risk for BED [57,62]. This issue is extremely delicate as, so far, interventions for prevention and treatment of being overweight/obese have mainly focused on dieting in order to restrict excess calorie intake [57].

Another important factor to consider is a sedentary lifestyle, a promoter of the overweight condition and critical in the course of BED patients with obesity [63]. This problem has been further enhanced in recent months because of the coronavirus disease 2019 (COVID-19) pandemic that has dramatically changed the daily routine of adolescents, with a large impact on their lifestyle and well-being [64,65]. The lockdown caused a marked decrease in PA levels [66] and increased occurrence of problematic eating behaviors among youths, leading to excessive weight gain, obesity development, and strong psychological distress [65,67].

In addition to risk factors, it is also worth examining the antecedents of a binge episode and the consequences. Among the antecedents it is possible to recognize behavioral and emotional factors [59]. Concerning the behavioral antecedents, consumption of “forbidden foods”—especially during a diet—and eating despite a lack of hunger have been reported [59,68]. Among emotional antecedents the most notable is eating in response to a negative emotion or trigger, such as shame, depression, anger, sadness, or guilt [41,59,61,68]. The consequences of a binge episode include both psychological and physical factors. Psychological factors include anxiety, guilt, shame and fatigue [59,68]; on the other hand, physical factors include feelings of fullness or sickness [59,68]. In addition to immediate consequences, binge eating was also significantly related to emotional/behavioral functioning, health-related quality of life, and physical dysfunctions in the long term [31,36,69,70,71]. Interestingly, Pasold et al. evaluated 102 adolescents that were overweight, aged 12–17 years, and analyzed their height, weight, and self-reported questionnaire data on emotional and behavioral functioning [69]. The study showed a strong positive correlation between BE, BMI, and depression, feelings of ineffectiveness, and negative self-esteem and significantly negatively related to somatic complaints and all aspects of health-related quality of life [69]. In the same study, BE was also correlated to fatigue and sleep difficulties [69]. Tanofsky-Kraff et al. analyzed the physical consequences of BE and obesity in the long term [71]. The researchers evaluated children aged 5–12 years at a high risk for adult obesity by giving them a questionnaire to assess binge eating at baseline and performing measurements of metabolic syndrome (MetS) components at baseline and at a follow-up 5 years later. Moreover, visceral adipose tissue (VAT) was measured with magnetic resonance imaging [71]. Children’s reports of BE predicted the development of MetS, correlated to increased triglyceride levels and VAT. These adverse metabolic health outcomes were partly correlated to the BE episode and the subsequent excessive weight gained [71]. The researchers thus concluded that BE may represent an early behavioral marker upon which to focus interventions for obesity and MetS [71].

## 5. Multidisciplinary Treatment of Binge Eating Disorder in the Adolescents

Adolescence represents a critical period for the onset of EDs, including BED [72]. During this period, neurobiological and body modifications may be accompanied by increased concern and attention to body size and shape [69,71]. As discussed above, BED diagnostic criteria are reported in the DSM-5. However, during adolescence the diagnosis of BED may present some difficulties owing to the growth phase, the consequently different energy requirements, and the ambiguous framework of the disease [28,71,72]. In fact, in adolescents, episodes of BE might occur less frequently and with less regularity than that specified in the DSM-5 [33,37,73].

Eating behavior is driven by homeostatic energy requirements, which are regulated by metabolic, hormonal, and neural pathways [74]. Several environmental and psychological factors in vulnerable youth—such as severe and chronic childhood stressors, parental over-focus on eating, weight and shape [45,75]—may influence energy intake and lead to different eating episodes [74]. Eating episode subtypes include: “binge-like eating”, characterized by high levels of LOC/self-reported overeating; “eating in the absence of hunger” (EAH); and “appetitive eating”, hedonic eating driven by the motivational or reinforcing properties of food, such as cravings and/or perceived palatability [74]. The LOC overeating is considered the most salient marker of BED, and its identification might be an opportunity to prevent BED, especially in early adolescence [33,72]. However, since LOC does not correspond to the full syndrome, BED onset is frequently underdiagnosed in adolescence [70,73,76].

As already discussed, binge/LOC eating and being overweight/obese are mutual risk factors [37], and BED is often associated with being overweight or obese [33,70,73]. Moreover, BED often coexists with other somatic and psychiatric comorbidities such as depression, emotional and personality disorders [37,73,77]. This highlights the relevance of a multidisciplinary approach to manage BED in adolescents that consists of a structured lifestyle therapy program combining a healthy meal plan, PA, and behavioral interventions [70,73,77,78].

Considering the dietary approach, although it has been shown in adults that total protein intake over a wide dose range (0.5–3.5 g/kg body weight) could increase lean body mass and facilitate loss of fat mass [79], conflicting results have been obtained regarding the association between protein intake after infancy and growth markers in late childhood and adolescence [80]. Furthermore, considering adolescents affected by obesity, studies in the literature [70,73,78], underline that dietary attitudes should be avoided as they have been associated with an increased risk of disordered eating and the development of ED [70,73,78]. Conversely, a regulated and balanced diet could reduce not only body weight but also BED symptoms [73,81].

Regarding PA, interventions should aim to decrease sedentary behavior and increase time spent in moderate-to-vigorous PA (60 min a day recommended) [81,82,83,84,85].

Regarding behavioral interventions, cognitive behavioral therapy (CBT), interpersonal psychotherapy (IPT), and dialectical behavioral therapy (DBT) have shown similar efficacy [82,85,86]. The aim is to assist subjects in modifying their dietary, PA, and sleep behaviors by controlling negative emotional states and thoughts [82,85,87]. Moreover, therapeutic intervention should also include family-based therapy, because an improved family environment can focus the treatment more on obesogenic eating behavior [27,82].

In light of all these components, the multidisciplinary approach should include an interdisciplinary team of experts including psychiatrists, pediatricians, nurses, a family therapist, a psychologist, registered dieticians, health coaches, exercise physiologists and others in order to be effective [70,73].

To date, various scientific societies have emphasized the importance of multidisciplinary treatment (MT) for the prevention of adolescent obesity and for health care [88,89,90]. There is a wealth of literature [91,92,93,94] focused on the effectiveness of MT in children and adolescents affected by obesity, particularly in reducing BMI and the risk of future comorbidities [83,95]. Conversely, despite the close relationship between obesity and eating disorders, only a few studies have considered the effect of MT on the development, progression, or reduction of eating disorder symptoms in children and adolescents that are overweight or obese [6,33,36,37,83].

In this regard, a recent systematic review [6] evaluated the impact of MT on ED symptoms in adolescents that are overweight or obese. The authors detected overall positive short- and long-term effects of MTs on ED symptoms, even though they were not always associated with BMI reduction [6]. In all selected studies, MTs included nutritional and lifestyle advice (structured dietary and physical interventions in some studies) and psychological treatments such as CBT, family-based intervention, and support group therapy [32]. Specifically, MT was found to be associated with a significant decrease in disinhibition of control and external and emotional eating [4]. This finding strengthens the conclusion that MT enables adolescents to react positively to emotional stress and external stimuli [6]. However, conflicting results were observed regarding dietary restraint.

Moustafa et al. [36] analyzed the association between MT and binge eating (BE) and LOC in youth with obesity in 29 studies. The overall results showed a significant decrease in BE and LOC after interventions [36]. Moreover, higher baseline BE and LOC measures appeared to prevent weight-loss success, and greater reductions in BE and LOC were associated with improved weight loss [36].

The meta-analysis of Jebeile et al. [83] assessed the impact of obesity treatment with a dietary component on ED and related symptoms in children and adolescents that were overweight or obese. It considered 36 studies with follow-up periods of 6 months to 6 years from baseline and showed a reduction in ED prevalence, risk, and symptoms [91]. In particular, a reduction in bulimic symptoms, emotional eating, binge eating, drive for thinness, and eating concerns was observed post-intervention and confirmed even at follow-up [83].

Most lifestyle intervention programs are focused on weight reduction as the primary treatment outcome. However, considering the bidirectional impact of obesity and BED in adolescents and the comorbidity presence of other psychiatric and somatic disorders [33,37], treatment programs for young people that are overweight or obese should consider a broader multidimensional approach. The measure of success of a lifestyle intervention for adolescents with BED who are affected by being overweight and obesity should be extended from mere weight reduction to behavioral and psychological improvements, including eating and dietary habits [31,78,83].

## 6. Positive Effects of Exercise in the Treatment of Binge Eating Disorder

Exercise, defined as “planned, structured, and repetitive and with a final or an intermediate objective improvement or maintenance of physical fitness”, is gaining more and more attention as a strategy to treat patients that are overweight who suffer from comorbid mental disorders and psychopathologic conditions, such as BED [82,96]. In the considered studies, exercise definition included aerobic exercise, high intensity, and mixed exercise (i.e., aerobic and resistance exercise).

Exercise programs, which are extremely useful as strategy to prevent and treat being overweight in children and adolescents [91,92,97], may be particularly appropriate for subjects with obesity and BED, thanks to their potential to improve both physical and psychological health simultaneously [82,98,99,100,101], (Figure 1). Indeed, when paired with structured dietary interventions, exercise has a strong positive impact on ED symptoms in adolescents that are overweight or obese [32]. Physical activity is instead defined as “any bodily movement produced by skeletal muscles that results in energy expenditure” [96]. In the studies considered, PA was performed at light to moderate and vigorous intensity. Concerning physical parameters, PA, both light-moderate and vigorous, has been correlated to BMI reduction, a decrease in blood pressure, lower levels of low-density lipoprotein cholesterol, and improved insulin resistance in adolescents diagnosed as overweight or obese [92,99,100,102,103,104,105]. Regarding psychological parameters, Daley et al., in a randomized controlled trial, evaluated 81 adolescents referred to a children’s hospital for obesity and, in addition to changes in BMI, they found changes in self-perception (self-esteem), depression, affect, PA, and aerobic fitness with exercise therapy [98]. Specifically, the exercise therapy consisted of a range of aerobic exercise activities (i.e., stepping, cycling, rowing, dance mat, and walking) and were performed intermittently at moderate intensity (40–59% of heart rate reserve) for 30 min, three times per week for 8 weeks (24 sessions) [98]. Importantly, their findings confirmed that both psychopathological conditions—a serious health concern in adolescents with obesity—and physical fitness improved upon supervised exercise therapy intervention [98]. The findings should be interpreted in light of several strengths and limitations [98]. The inclusion of both exercise placebo and usual care groups is a significant advancement with respect to previous research. Moreover, the authors recorded a high rate of recruitment of eligible patients via pediatrician referral (48%). Among the strengths, it is worthwhile underlining that different studies evaluating lifestyle treatments for child obesity tended to recruit mainly white children, limiting their generalizability. In contrast, Daley et al. recruited a more racially diverse population from a wide range of socioeconomic status categories [98]. Unfortunately, blinding of the assessments was not done, and this may be a possible limitation. Moreover, the possibility of a type 1 error attributable to multiple statistical testing exists, and thus the noted differences may be spurious. The consistent trend toward benefit for exercise anyway suggests that the differences identified are real [98].

Regular PA, from light to vigorous, was found to alleviate symptoms of anxiety and depression [96]. Moderate intensity PA has also produced positive cognitive effects by improving eating behavior and self-regulation in healthy students [106], and non-compensatory PA has been associated with less psychopathology in patients with BED or bulimia nervosa [107]. Limitations of the studies, however, should be recognized when interpreting the findings: indeed, the works relied on self-reported PA data, which may be biased or unreliable. Moreover, the cross-sectional nature of the research precludes causal interpretations [107].

In a review analyzing the relationship between exercise and PA in the prevention and treatment of different mental disorders in the adult population, Zschucke et al. reported that PA was essential in BED, as most patients tended to not exercise at all. Specifically, the authors reported that moderate exercise intervention (walking) was associated with reduced weight, depression scores, and, when paired with cognitive behavioral therapy (CBT), binge episodes [108,109,110]. In line with these outcomes, Ashdown-Franks et al. and Blanchet et al. reported that exercise intervention was correlated with decreased frequency of BE episodes and a significant decrease in depressive symptoms in patients with BED [111,112]. The diversity of these studies, the use of self-report measures and the lack of blinding evaluations are the main limitations of this research.

Both aerobic and resistance exercise delivered over a median of 7 weeks (30–90 min, three times a week) are reported to have positive effect on symptoms of depression in adolescents [113]. In addition, it was reported that once weekly, 60 min aerobic exercise program was associated with less BE in young women with BED [108]. Moreover, Vancampfort et al. highlighted the reduced eating pathology in adolescents and young adults with EDs, affirming that aerobic exercise, yoga, and basic body awareness therapy might improve the mental and physical quality of life in patients with an eating disorder [113] by reducing both binge eating and body weight [113,114]. The paucity and heterogeneity of available studies limit overall conclusions of this systematic review and highlight the need for further research. In light of these promising results, Mathisen et al. compared the effects of physical exercise and dietary therapy (PED-t) to CBT in the treatment of bulimia nervosa and BED in young adults and the adult population [115,116]. Specifically, the exercise program proposed consisted of three weekly exercise sessions, each of 40–60 min duration. Two sessions were supervised resistance exercise, the third one instead was unsupervised pyramid interval running, involving shifts between intensive work-periods and active rest-periods with progressive duration (from one minute work period to 4 min of work period) [115]. Among outcome measures were the Eating Disorder Examination–Questionnaire (EDE-Q), Satisfaction with Life Scale (SWLS), and Beck Depression Inventory (BDI) [115,116]. Both PED-t and CBT resulted in significant improvements on all outcomes, with a long-term effect; 30–50% of participants positively responded to treatments, with no statistical difference between the two groups [115,116]. Thus, the researchers found support for their hypothesis, as the PED-t and CBT performed equally in alleviating the symptoms of bulimia nervosa and BED and in improving well-being and psychosocial impairment [116]. In addition, PED-t partly attenuated symptoms of depression [116]. Alleviations of core ED symptoms is fundamental for a treatment to be successful, but improvements in secondary outcomes are equally important to consolidate symptom improvements [116].

The limited number of studies of patient satisfaction with treatment have used biased retrospective recalls of treatment modalities rather than methods [115]. In contrast, Mathisen et al. performed prospective quantitative and qualitative measures to study how satisfaction might be associated with pretreatment expectations, treatment elements, or generic factors in the two different treatment arms. This is an important strength of their work [115,116].

Concerning the type of proposed intervention, a specific more effective exercise modality has been not univocally defined in BED treatment, but it appears that low intensity exercise (e.g., yoga or walking between one to three times per week) is enough to exert a positive impact on BED [112]. Galasso et al. evaluated the effects of a specific training in addition to conventional treatment of eating disorder symptoms, anthropometric characteristics, and physical performance in 19 women affected by BED [117]. The sample was divided in two groups: 10 women carried out Combined Aerobic and Anaerobic Exercise Training (CAAET) in addition to conventional treatment (diet plus CBT), whereas the remaining nine followed the conventional treatment alone [117]. After six months of treatment, a significant decrease in binge episodes, weight, and BMI, and an increase in exercise capacity was observed in both groups [117]. Moreover, the CAAET group presented a greater improvement in aerobic performance, evaluated with Six-Minute Walking Test, than that observed in the control group [117]. Although both interventions similarly improved BED symptoms, adding PA in the treatment could enhance the long-term maintenance of both weight loss and reduction in binge episodes in these patients [117]. The major limitation of this study was the short duration of CAAET. However, the short duration of CAAET was shown to be sufficient to improve anthropometric parameters, ED symptoms and exercise conditions [117]. Higher-powered confirmatory PA programs are needed to understand both the types and doses of exercise and the effects of PA in these patients. In addition, future research is needed on a large sample size, with more diversity in age range (especially including pediatric patients) and inclusion of both women and men [117].

The effect of PA on body composition might also be influenced by the intensity level: high-intensity exercise favors more negative energy and metabolic adaptations in the muscle than lower-intensity exercise [112]. Unfortunately, the evidence regarding the mechanisms behind the association between PA and BED in adolescents is scarce, but the potential underlying mechanisms of PA in BED patients were explored in an interesting systematic review of the adult population [112]. In the review, different types and levels of PA were evaluated, from 30 min of aerobic exercise 3 to 5 times a week, to 1 h of yoga once a week plus daily 30-min session of home-based exercises [112]. One potential mechanism is related to the influence of PA on the reward system. Indeed, food is considered a natural reward that can be influenced by PA [112]. Evidence in young adults shows that PA helps decrease cravings and that food addiction and BE share similar pathways, thus supporting this mechanism [112,118]. Another mechanism seems to be the effect of PA on the negative effect: it was shown that exercise influences the neurotransmitters modulating mood, such as serotonin and endorphin secretion [119]. PA promotes a reduction of stress and anxiety and improves mood through the hypothalamus–pituitary–adrenal axis [112,120]. As suggested by Zschucke et al., PA could reduce food cravings because stress and anxiety are known triggers of BE episodes [121]. Thus, PA has an indirect effect on BE through its influence on mood, stress, and anxiety [112,121]. Another potential mechanism correlating exercise and BED is related to appetite control. Among the exercise-induced physiological processes influencing appetite are the stimulation of gastric emptying and the increase in appetite-related peptides (such as peptide tyrosine-tyrosine and glucagon-like peptide-1) [122]. Moreover, constant PA (which includes both aerobic and resistance exercises) affects body composition, leading to changes in resting metabolic rate, influencing daily energy intake, promoting BMI reduction, and improving MetS [112,123]. Interestingly, the optimal timing of exercise to decrease BE episodes has been hypothesized: PA could have a positive effect if performed before the BE episodes or during cravings in order to replace the BE episodes or reduce the caloric intake [112,124]. Lastly, evidence indicates that PA improves sleep, which is disturbed in adolescents with EDs and correlated with increased risk of weight gain and cardio-metabolic dysfunction in obese adolescents [125,126,127,128,129].

In contrast with previous studies, Martinez-Avila et al. evaluated the relationships between the time spent in sedentary behavior and PA of different intensity, assessed with a wrist-worn GT3X+ model accelerometer (ActiGraph, Pensacola, FL, USA) for seven consecutive days (24 h/day), and eating behavior traits in young, healthy adults [130]. The researchers reported that BE and emotional eating, examined via the self-reported questionnaires (Binge Eating, Three-Factor Eating Questionnaire-R18 and Control of Eating Questionnaire) were inversely associated with time spent in sedentary behavior and directly associated with PA in young adults [130]. The present work suffers from the possible limitation that the paper-based questionnaires were assessed during visits in the presence of a nutritionist whom the subjects may have tried to please, introducing a certain bias into their answers. In addition, the patients saw the questionnaires twice, once at baseline and again after the intervention and this may have introduced some ‘learning’ bias. Further research is thus needed to reach conclusive results concerning this important theme.

In their review, Cook et al. summarized divergent studies and identified specific guidelines that may enhance ED treatment outcomes through PA [131]. The practical set of guidelines provided by the authors for the clinical management and therapeutic use of exercise in ED treatment focus on empowering ED patients with exercise as a tool for healthy living [131]. Specifically, the review highlighted the importance of a multidisciplinary approach, with a team of experts in exercise, nutrition, medicine, mental health, and physical therapy who develop individually tailored programs [131]. Moreover, it is fundamental to identify patients at risk for pathological attitudes and behaviors toward exercise (e.g., exercise dependence and compulsive exercise) in which PA may exacerbate ED pathology [131]. Importantly, the correct modality and amounts of exercise must be evaluated according to the physiological and psychological needs of the patient; for instance, aerobic activity may promote weight loss and reduce bulimic symptoms and body dissatisfaction in individuals with bulimia nervosa and BED [131].

Unfortunately, several limitations are present this review and, in the guidelines proposed by the authors. First, there is a lack of consensus on exactly which exercise behaviors correspond to specific outcomes in each ED variant. Second, no specific guidelines tailored for specific outcomes were provided. Third, although several RCTs and meta-analysis were evaluated in the study, there are relatively few large-scale, high-quality RCT that have examined the use of exercise in ED treatment.

It is worth to mention that an effective strategy to prevent obesity development in adolescents and children is to lead an active life, with daily light intensity PA, while sedentary behavior is considered a risk factor both for obesity and for BE episodes [81,82,83,85,132]. Lastly, in the recent years, scientific evidence has shown that resistance exercise has many beneficial health effects, as reducing cardiac adipose tissue mass and the consequence risk of cardiovascular adverse events [133]. Resistance training has also been associated with reduced body fat percentage, body fat mass and visceral fat in young adults [134]. This type of exercise, differently from other exercise modalities as running or jumping, can be applied to obese adolescents without producing musculoskeletal problems, that could be further enhanced by excess body weight [129,130]. Thus, additional studies on resistance training in obese adolescents with comorbid BED would be of great interest.

**Figure 3:**
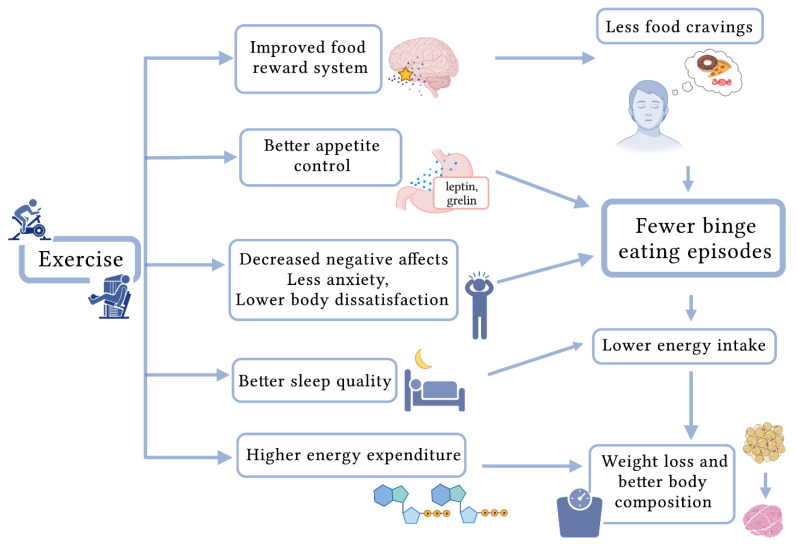
Physical and psychological effects of exercise programs as a preventive and therapeutical strategy for binge eating in adolescents with obesity.

In Figure 3, physical and psychological effects of exercise programs as strategic approach for binge eating in adolescents with obesity are reported.

## 7. Conclusions

Binge ED and obesity are strictly correlated. In youth, the two conditions share risk factors and consequences at both the physical and psychological levels, requiring special care [31,57]. Specifically, BED is a risk factor for obesity in childhood and adolescence [37,40,41] and being overweight or obese may increase the risk for BED, as binge eating is more likely to be associated with populations where individuals are on average overweight [37,42].

Considering this, it might be fundamental for an early identification of the patients at risk for pathological attitudes and behaviors to prevent the EDs. Further research is needed to reveal all the possible actors of such disorders, including nutrigenetic factors, that may play a key role not only in etiopathogenesis but also in novel integrated therapeutic approaches to limit obesity pandemic and decrease BED complications.

Until now, with the scientific evidence provided, a tailored and integrated multidisciplinary treatment is critical to provide adequate care of patients with obesity and ED, to address their excess body weight, BED, and the consequences [36,57,59]. This multidisciplinary approach should combine a structured lifestyle therapy program combining a healthy meal plan, PA, and behavioral interventions, involving interdisciplinary team of experts [73].

Even though additional research is needed to reach conclusions about the role of exercise in the treatment of obesity and comorbid BED, especially in adolescents, promising results have already suggested that closely monitored exercise is safe and, paired with CBT, may provide multiple benefits on both the physical and the psychological levels. Integrated treatment programs addressing weight management and eating disorders and including exercise programs for individuals with obesity are necessary to provide tailored and timely treatment and prevent severe complications.

There are several limitations in our study. Most MTs in literature focus on weight reduction as a primary outcome of treatment rather than behavioral and psychological improvements. Furthermore, accurate information concerning duration, frequency, intensity, or type of exercise to be used in these patients are often absent.

Existing studies on PA as an add-on to CBT in ED patients are scarce and those analyzing BED patients have limitations, such as relying on subjectively assessed PA (instead of objective indicators) or assessing only physical symptoms rather than ED symptoms. Another limit may be the lack of the blinding of participants and personnel in interventions involving PA. Moreover, the heterogeneity of interventions (in terms of intensity, modality, frequency, and duration of exercise) and the paucity of studies limit overall conclusions. Lastly, studies using a cross-sectional design, as observational studies, cannot be regarded as indicators of a causal relationship between PA and BED improvement.

Thus, we suggest that the measure of success of a multidisciplinary intervention for adolescents with BED who are affected by being overweight and obese should consider not only weight reduction, but also behavioral and psychological improvements. Moreover, the correct way to exercise should be considered according to physical and physiological needs of the subject. Future research must concentrate on the physical and psychological effects of specific forms of exercise, also evaluating the possible role of resistance training.

Even though additional research is needed to reach definitive conclusions about the role of PA in the treatment of obesity and comorbid BED, especially in adolescents, promising results have been suggested. Tailored and multidisciplinary treatments both for the management of weight and for eating disorder symptoms are important to treat adolescents promptly and effectively for obesity and BED.

## Data Availability

Not applicable.
